# Melt-Spun Fibers from Bio-Based Polyester–Fiber Structure Development in High-Speed Melt Spinning of Poly(ethylene 2,5-furandicarboxylate) (PEF)

**DOI:** 10.3390/ma14051172

**Published:** 2021-03-02

**Authors:** Wataru Takarada, Kenichi Sugimoto, Hajime Nakajima, Hendrikus A. Visser, Gert-Jan M. Gruter, Takeshi Kikutani

**Affiliations:** 1 Department of Materials Science and Engineering, Tokyo Institute of Technology, 2-12-1 O-okayama, Meguro-ku, Tokyo 152-8550, Japan; takarada.w.aa@m.titech.ac.jp; 2 Reinforcement Materials Research Department, Bridgestone Corporation, 3-1-1, Ogawahigashi-cho, Kodaira-shi, Tokyo 187-8531, Japan; kenichi.sugimoto@bridgestone.com; 3 Avantium Renewable Polymers BV, Zekeringstraat 29, 1014 BV Amsterdam, The Netherlands; info@avantium.com (H.N.); roy.visser@avantium.com (H.A.V.); gert-jan.gruter@avantium.com (G.-J.M.G.)

**Keywords:** bio-based polyester, poly(ethylene 2,5-furandicarboxylate), high-speed melt spinning, molecular orientation, orientation-induced crystallization, mechanical properties

## Abstract

Poly(ethylene 2,5-furandicarboxylate) (PEF) is regarded as a bio-based alternative or complementary polyester for the widely used fossil resource-based polyester, poly(ethylene terephthalate) (PET). High-speed melt spinning of PEF of low and high molecular weight (L-PEF, H-PEF) was conducted, and the structure and properties of the resultant as-spun fibers were investigated. The occurrence of orientation-induced crystallization was confirmed for the H-PEF at the take-up velocity of 6.0 km/min, the highest speed for producing PET fibers in the industry. Molecular orientation and crystallinity of the as-spun fibers increased with the increase of take-up velocity, where the H-PEF fibers always showed a higher degree of structural development than the L-PEF fibers. The tensile modulus of the high-speed spun H-PEF fibers was relatively low at 5 GPa, whereas a sufficiently high tensile strength of approximately 500 MPa was measured. These values are adequately high for the application in the general semi-engineering fiber field.

## 1. Introduction

Poly(ethylene 2,5-furandicarboxylate) (PEF) is a promising plastic for a wide range of general and semi-engineering applications, by utilizing its attractive physical properties and functionalities. The generally known interesting physical properties of PEF compared to conventional-polyesters such as poly(ethylene terephthalate) (PET) and poly(trimethylene terephthalate) (PTT) are higher glass transition temperature (T_g_), modulus, and tensile yield strength [1]. The high gas barrier is one of the most promising functionalities of PEF. The gas barrier of PEF to O_2_, CO_2_, and H_2_O are 6–11, 8–19, and 2–3 times higher than that of PET, respectively, and these are featured for many packaging applications [2,3,4,5].

The recent progress in the bio-based production of 2,5-furandicarboxylic acid (FDCA) brings more advantages for PEF. The production of FDCA (C_6_H_4_O_5_) from abundant C6 sugars (e.g., fructose and glucose; C_6_H_12_O_6_) is more favorable than the bio-based production of terephthalic acid (TPA), as C6 sugars to C8 TPA is stoichiometrically and hence techno-economically unfavorable compared to C6 to FDCA [6,7]. As this sustainable and economic advantage of FDCA is also applicable to the petroleum-based polyesters such as poly(trimethylene 2,5-furandicarboxylate) (PTF) versus bio-based PTT, and poly(butylene 2,5-furandicarboxylate) (PBF) versus bio-based poly(butylene terephthalate) (PBT). FDCA based polymers have tremendous potential to transition the existing polymer industry into a more environmentally friendly one.

In fact, PEF is close to creating a ‘new plastic economy’, especially in the film and bottle industry. Some of the excellent properties (Tg, barrier, optical properties, surface energy, and mechanical performance) are specifically useful in film and bottle applications. PEF is currently being developed for multilayer and monolayer barrier (shrink) film in packaging as well as display and electronic applications, as a barrier layer in multilayer bottles, for multi-use bottles, and for single layer (small) bottles for carbonated soft drinks (CSD), juices, water and beer. For the multilayer applications the compatibility of PEF (used as a barrier layer) in the PET recycling stream is a clear value proposition compared to incumbent barrier materials.

The technical development of film and bottle applications resulted in interesting scientific development for PEF such as crystallization kinetics [8,9,10,11,12], nano-ordered morphologies, and its thermal ageing effect [8,13]. Many of the unique properties can be explained by the unique chain mobility of PEF which results from the hindrance of furan-ring-flipping in PEF due to ring polarity and structural non-linearity [14,15,16]. For the drawing behavior, the lower entanglement density of PEF plays a pivotal role, leading to a later onset of strain induced crystallinity upon stretching in the rubbery phase when compared to PET [17]. 

If we aim for more proactive growth of PEF in the industry of polyesters, the potential of application possibilities of PEF in fiber applications should be discussed. This requires more insight into fiber spinning and fiber properties. A good spinnability of PEF can be predicted as PEF intrinsically has the appropriate crystallization kinetics, good and fitting rheology [17], and the right thermal properties and behavior. To the best of the authors knowledge only limited data is available in scientific literature on the processing of PEF into fibers and their performance. In a broad study on the filament extrusion of biobased polymers a scoping trial on producing PEF fibers and their basic performance was reported [18]. Here, the PEF partially oriented yarn (POY) produced show a tenacity close to that of PET, but with a lower value of the elongation at break. In a study on the influence of poly(ethylene glycol) modified PEF, Ji et al. presented fiber spinning data and main fiber properties of PEF. [19]. Here, a low tenacity is reported for the PEF (1.15 cN/dtex), alongside a remark that the PEF monofilament was difficult to draft and behaved in a brittle manner. This was possibly related to the molecular weight of the final filaments, however, only the intrinsic viscosity of the PEF resin used was reported (0.61 dL/g). For large scale application of PEF in fibers, the development of a high-speed PEF spinning process is highly preferable. In this study, a typical high-speed spinning process which was originally designed for PET is applied to PEF, with a focus on the spinnability and the different characteristics of PEF fibers versus PET fibers.

## 2. Materials and Methods 

### 2.1. Preparation of Fibers

#### 2.1.1. Highspeed Melt Spinning

Two types of PEF with different molecular weight were used in this study. Both PEF batches were polymerized in the melt state using 2,5-furandicarboxilic acid (FDCA) produced using the YXY technology and monoethylene glycol. Subsequently, both resins underwent solid state polymerization in a tumble dryer under vacuum for a different duration resulting in a difference in their final molecular weights. The first one is low molecular weight PEF, which is abbreviated by L-PEF (number average molecular weight (Mn) of 35,200, weight average molecular weight (Mw) of 75,000, and intrinsic viscosity (IV) of 0.80 dL/g). The second one is high molecular weight PEF (Mn of 44,000, Mw of 106,100, and IV of 1.05 dL/g). This high molecular weight PEF is abbreviated by H-PEF. The molecular weights were determined using gel permeation chromatography with a classical calibration to polystyrene standards in a mixture of 40 wt. % 2-chlorophenol and 60 wt. % chloroform. The intrinsic viscosity was determined according to ASTM D4603 using a mixture of 60 wt. % phenol and 40 wt. % 1,1,2,2-tetrachloroethane at 30 °C. Both L-PEF and H-PEF were supplied by Avantium, Amsterdam, Netherlands.

High-speed melt spinning of the PEF polymers was carried out using an extrusion setup consisting of a single-screw extruder, a gear pump and a spinning head. A spinneret with a single hole of diameter 1.0 mm and L/D of 2.0 was attached to the spinning head. The PEF polymers were extruded with a throughput of 5.0 g/min for the L-PEF and 3.0 g/min for the H-PEF. The extrusion temperature was set to 275 °C for the L-PEF and 320 °C for the H-PEF. The selection of different extrusion conditions for the two types of PEF was done on the basis of the optimization of extrusion behavior by tuning torque and pressure. There can be a certain degree of thermal degradation especially for H-PEF with the higher extrusion temperature and lower throughput, i.e., longer residence time at higher temperature in the extrusion system. Therefore, the molecular weight was measured after the melt spinning. As a result of the melt spinning step, the Mw of the L-PEF has dropped from 75,000 to 52,500 and the H-PEF from 106,100 to 84,000. A take-up winder was set at 3.3 m below the spinning head. The take-up velocity varied from 1.0 to 6.0 km/min for both L-PEF and H-PEF.

On-line measurement of the spin-line diameter was carried out using a diameter monitor (Zimmer OHG, Model 460-A/10, Rheinau, Germany). The measurement was conducted from 10 to 300 cm down from the spinneret with an interval of 10 cm.

#### 2.1.2. Drawing and Annealing 

To discuss the characteristics of high-speed spun PEF fibers, structure of the PEF fibers prepared through the conventional in-line drawing process with and without applying the final annealing roll will be presented. The drawn fibers were prepared from H-PEF with the take-up velocity of 0.3 km/min, in-line drawing temperature and draw ratio of 90 °C and 4.5×, respectively, and final annealing temperature of 130 °C.

### 2.2. Characterization of the Obtained Fibers

#### 2.2.1. Birefringence 

The measurements of refractive indices of each fiber in parallel and perpendicular directions to the fiber axis (*n_/_*_/_ and *n*_⊥_) were carried out using an interference microscope (Carl-Zeiss Jena, Germany) equipped with a polarizing filter [20,21]. Birefringence, *Δn*, and Lorentz-density, *LD*, were calculated from the two refractive indices using the following Equations (1) and (2).

(1) $$\Delta n=n_{//}-n_{⊥},$$

(2) $$LD=\frac{{\bar{n}}^{2}-1}{{\bar{n}}^{2}+2},$$

where

(3) $${\bar{n}}^{2}=\frac{n_{//}^{2}+2n_{⊥}^{2}}{3}.$$

The birefringence represents the molecular orientation of the fibers. The Lorentz-density has a linear relation with density, and therefore represents the crystallinity of the fibers.

#### 2.2.2. Tensile Test

The stress–strain curve of the PEF fibers was measured using a tensile testing machine (SHIMADZU AG-I, Kyoto, Japan). The gauge length was 20 mm and the crosshead speed was set to 20 mm/min. Tensile modulus, tensile strength, and elongation at break were obtained by averaging the results of 10 trials of the tensile test of the samples. 

#### 2.2.3. Wide Angle X-ray diffraction (WAXD)

The crystalline structure of the as-spun fibers was investigated with the use of two-dimensional wide-angle X-ray diffraction (WAXD) pattern measurements. The WAXD intensity distribution measurement for the fiber bundles was performed using a nickel-filtered CuKα radiation source generated at 60 kV–45 mA and a mercury charged-coupled device (CCD) X-ray detector (Rigaku Co., Ltd., Tokyo, Japan) with a sample to CCD distance of 35 mm.

## 3. Results and Discussion

### 3.1. Diameter Profile of Spin-Line

The high-speed spinning was successfully performed and continuous PEF fibers were obtained. For H-PEF, occasional spin-line breakage occurred at the take-up velocities of 5.0 and 6.0 km/min. The fixed throughput of 5.0 g/min for L-PEF and 3.0 g/min for H-PEF was applied for all the take-up velocities, and this resulted in the different diameters of the as-spun fibers.

**Figure 1:**
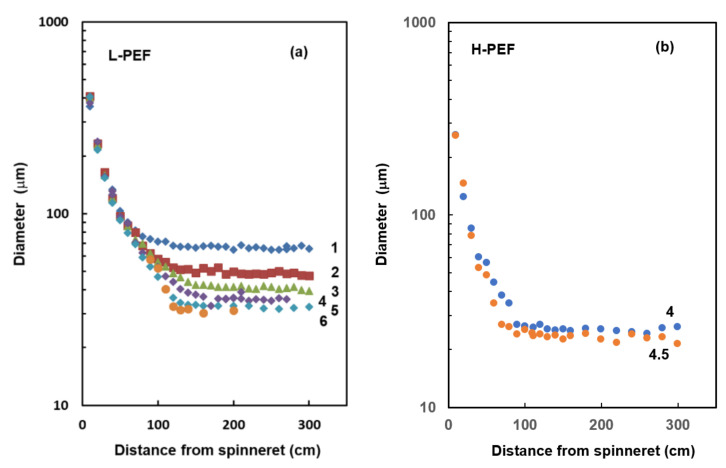
Diameter of (**a**) low molecular weight poly(ethylene 2,5-furandicarboxylate) (L-PEF) and (**b**) high molecular weight PEF (H-PEF) spin-line versus the distance from the spinneret. Take-up velocity (km/min) is indicated in the figure.

Results of on-line diameter measurement for the spin-line of L-PEF at different take-up velocities are shown in Figure 1a. The thinning behavior was not affected by the take-up velocity down to the position of about 50 cm from the spinneret. Below this position, the diameter profiles for different take-up velocities split, and there was a rough tendency that the spin-line diameter was lower with the increases to take-up velocity. Regarding the diameter profile of the spin-line, at the take-up velocities of 1.0 or 2.0 km/min, the spin-line diameter reduced smoothly and reached the final value asymptotically at the position of around 150–200 cm from the spinneret. When the take-up velocity was increased to 3.0 or 4.0 km/min, thinning of the spin-line tended to concentrate to the downstream, i.e., the region immediately above the solidification point. At the take-up velocity of 5.0 or 6.0 km/min, the downstream localization of deformation was enhanced and the position of the solidification apparently shifted to the upstream at around 120 cm from the spinneret. This behavior can be regarded as the occurrence of neck-like deformation, which has been observed in the high-speed melt spinning process of various crystalline polymers [22,23,24]. We found that the change in the thinning behavior of the spin-line with increasing take-up velocity resembled the one for the high-speed spinning of high molecular weight poly(ethylene terephthalate) [25].

As for the H-PEF, because of the limited availability of the PEF polymer along with the occasional breakage of the spin-line, we started the diameter measurement from 4.0 km/min with the intention of confirming the existence of neck-like deformation. The thinning behavior could be measured only at 4.0 and 4.5 km/min. The result is shown in Figure 1b. Existence of the neck-like deformation was observed clearly at around 90–100 cm. In other words, the neck-like deformation for the H-PEF started to occur at a lower take-up velocity in comparison with the L-PEF even though the extrusion temperature was raised from 275 to 320 °C. This was obviously due to the higher viscosity of H-PEF. A similar observation was reported by Dolmans [18], where the extrusion temperature as well as the maximum pressure during extrusion was increased to deal with the higher viscosity of PEF. The lower through-put of the H-PEF of 3 g/min versus 5 g/min for the L-PEF can be another reason for the enhancement of the neck-like deformation. 

**Figure 2:**
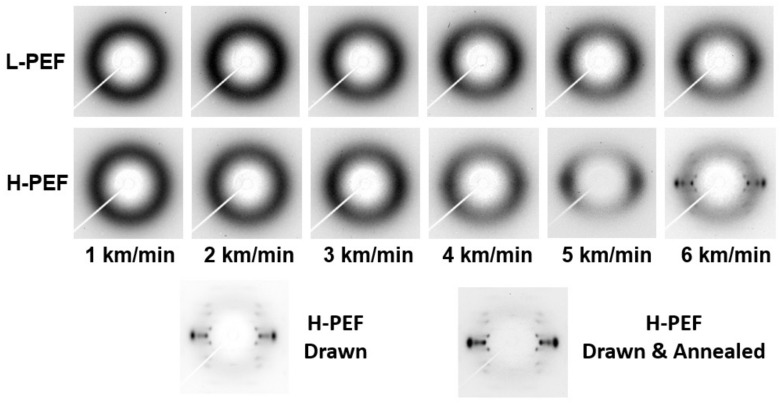
Wide-angle X-ray diffraction (WAXD) patterns of high-speed spun PEF fibers obtained at various take-up velocities. WAXD patterns of drawn fiber and drawn and annealed fiber are also shown for comparison.

The two-dimensional wide-angle X-ray diffraction (WAXD) patterns of the as-spun fibers are shown in Figure 2. The WAXD patterns of the drawn fiber and the drawn and annealed fiber are also shown for comparison. Only the isotropic amorphous halo was observed for the high-speed spun fibers prepared at the low take-up velocity of 1.0 or 2.0 km/min. The intensity of amorphous halo gradually concentrated to the equator with the increase of take-up velocity, where the H-PEF fibers showed a higher degree of anisotropy than the L-PEF fibers. The distinct crystalline reflections of high orientation with the alignment of crystallographic *c*-axis parallel to the fiber axis appeared only for the H-PEF fiber prepared at 6.0 km/min. It should be noted that the occurrence of neck-like deformation in the spinning process does not necessarily correspond to the appearance of distinct crystalline reflections in the WAXD measurement. Similar behavior was found in the high-speed melt spinning of semi-aromatic polyester with higher rigidity of molecular chain than PET, i.e., poly(ethylene 2,6-naphthalene dicarboxylate) [26]. The diffraction pattern was similar with those of the drawn fibers and the drawn and annealed fibers, however, a relatively strong amorphous halo still existed in the WAXD pattern of 6.0 km/min fiber.

**Figure 3:**
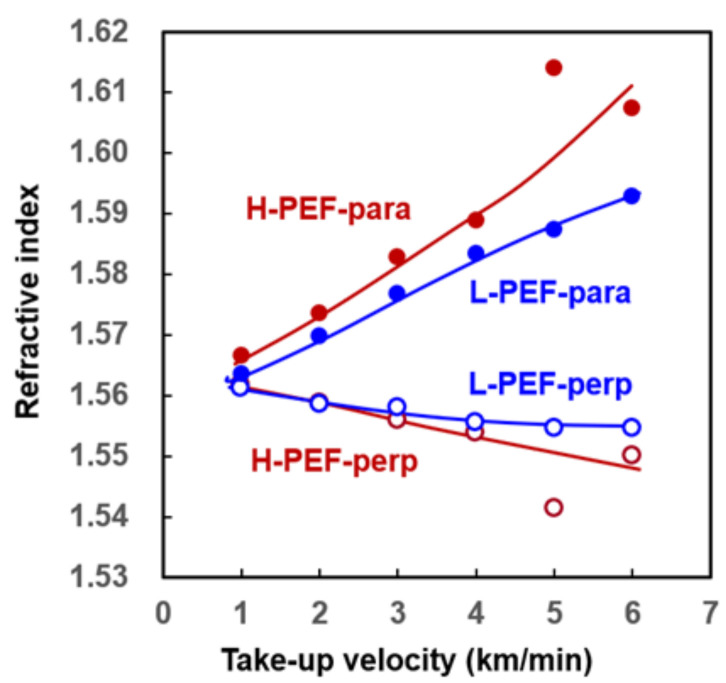
Variation of refractive indices in the directions parallel and perpendicular to the fiber axis with take-up velocity for high-speed spun L-PEF and H-PEF fibers.

The variations of refractive indices in the parallel and perpendicular directions to the fiber axis, *n*_//_ and *n*_⊥_, with the take-up velocity are shown in Figure 3. Both for the L-PEF and H-PEF, there was an increase of *n*_//_ and a decrease of n_⊥_ with the increase of take-up velocity. It is known that various high-speed spun fibers exhibit a similar trend [27]. When compared at the same take-up velocity, the H-PEF fibers showed the higher *n*_//_ and lower *n*_⊥_ in comparison with the L-PEF fibers.

It should be noted that the data for H-PEF prepared at 5.0 km/min showed extremely high *n*_//_ and low *n*_⊥_. Diameter of the fiber was slightly higher in comparison with the value predicted from the through-put and take-up velocity. Other data for this fiber, which will be presented later, also appear to be slightly odd in comparison with other fibers. We need to admit here that there could be some unknown problems in the stage of preparing this particular fiber. As mentioned in Section 3.1, because of the limitation of the amount of H-PEF available, we could not retest the melt spinning experiment. At this moment, we think that even though the data is doubtful, there still is a possibility that these data are correct. One reason is that we found similar behavior in the high-speed melt spinning of poly(phenylene sulfide), in which there was a hesitation of birefringent increase and an abrupt reduction of tensile strength of as-spun fibers when orientation-induced crystallization started to occur with the increase of take-up velocity [28].

From Figure 3, it also can be seen that the average refractive index of PEF estimated based on Equation (3) is around 1.56–1.57, while the refractive index of PET is known to be around 1.58–1.60 [27]. According to the Lorentz–Lorenz equation, refractive index becomes higher for the polymer with the higher polarizability of repeating unit and higher density. Density of PEF is higher than that of PET. Considering the similarity of chemical structure between PEF and PET, it can be concluded that the furan ring has lower polarizability than the benzene ring.

**Figure 4:**
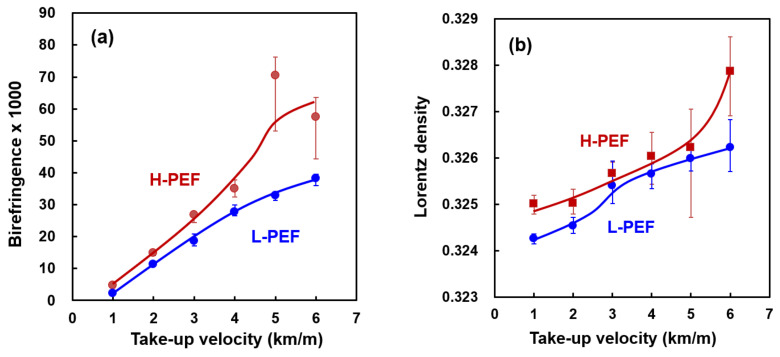
Variations of (**a**) birefringence and (**b**) Lorentz density with take-up velocity for high-speed spun L-PEF and H-PEF fibers.

Birefringence was calculated as the difference of *n*_//_ and *n*_⊥_. As explained in the experimental section, birefringence represents the degree of molecular orientation. Figure 4a shows a plot of birefringence of the as-spun L-PEF and H-PEF fibers against the take-up velocity. The birefringence of PEF fibers increased with the increase of take-up velocity, where the H-PEF fibers showed higher birefringence than the L-PEF fibers at all the take-up velocities. It should be noted that the birefringence increased almost linearly with the take-up velocity, while the birefringence of high-speed spun PET fibers is known to show sigmoidal increase with the increase of take-up velocity [29]. If the stress-optical rule is applicable for the development of birefringence, birefringence is supposed to show a parabolic increase with the take-up velocity in the high-speed melt spinning process. This result may reflect the higher rigidity of the PEF molecules in the molten state.

Lorentz density varies with the mean refractive index, and has a linear relation with the density. Therefore, Lorentz density can be regarded as a measure of the crystallinity of fibers. The variation of Lorentz density of high-speed spun fibers with the take-up velocity is shown in Figure 4b. The Lorentz density increased with the increase of take-up velocity, and the H-PEF fibers showed higher Lorentz densities than the L-PEF fibers. It should be noted that the H-PEF fiber prepared at 6 km/min showed exceptionally high Lorenz density. This result may correspond to the appearance of crystalline reflections in the WAXD measurement shown in Figure 2. Even though there was no clear indication of the crystallization in the WAXD measurement for the fibers prepared at lower take-up velocities, density of the PEF fibers increased with the increase of molecular orientation. A similar trend was found for the high-speed spun fibers of poly(ethylene 2, 6-naphthalene dicarboxylate) [26].

**Figure 5:**
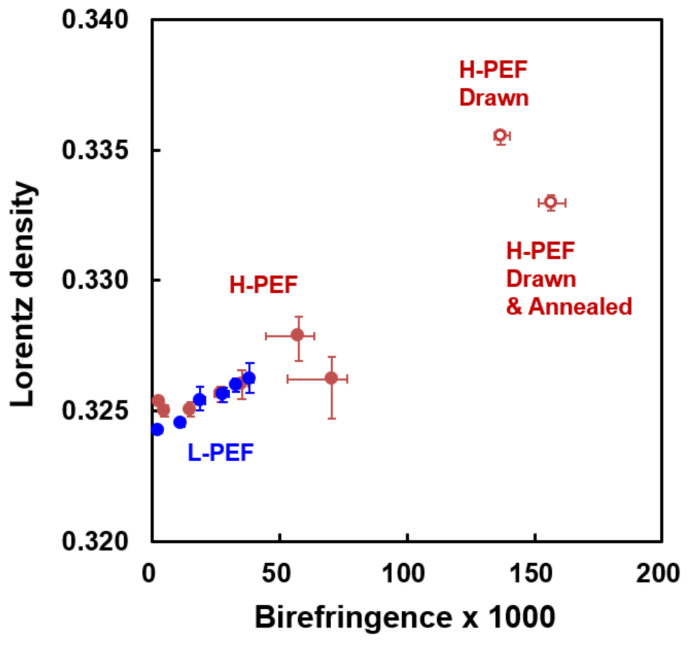
Relation between birefringence and Lorentz density for the high-speed spun L-PEF and H-PEF fibers. The results for the drawn and the drawn and annealed fibers are also shown for comparison.

Birefringence was plotted against Lorentz density as shown in Figure 5. In this figure, data for the drawn fibers and the drawn and annealed fibers are shown for comparison. Birefringence and Lorenz density showed an almost linear relationship. It was found that the degree of structure development of high-speed spun fiber is still at a middle stage in comparison with the structure of fully drawn and annealed fibers. The increase of the Lorentz density without the appearance of distinct crystalline reflections for the high-speed spun PEF fibers may suggest the development of structural ordering in the amorphous phase with the increase of molecular orientation.

The annealing step lowers the Lorentz density, indicating formation of crystals from the oriented amorphous regions does not promote the lateral packing of molecular chains and even reduces the structural ordering. In spite of this, the annealing step slightly increases the birefringence at the same time indicating an increase in the overall molecular orientation in the fiber.

### 3.2. WAXD Intensity Profile

**Figure 6:**
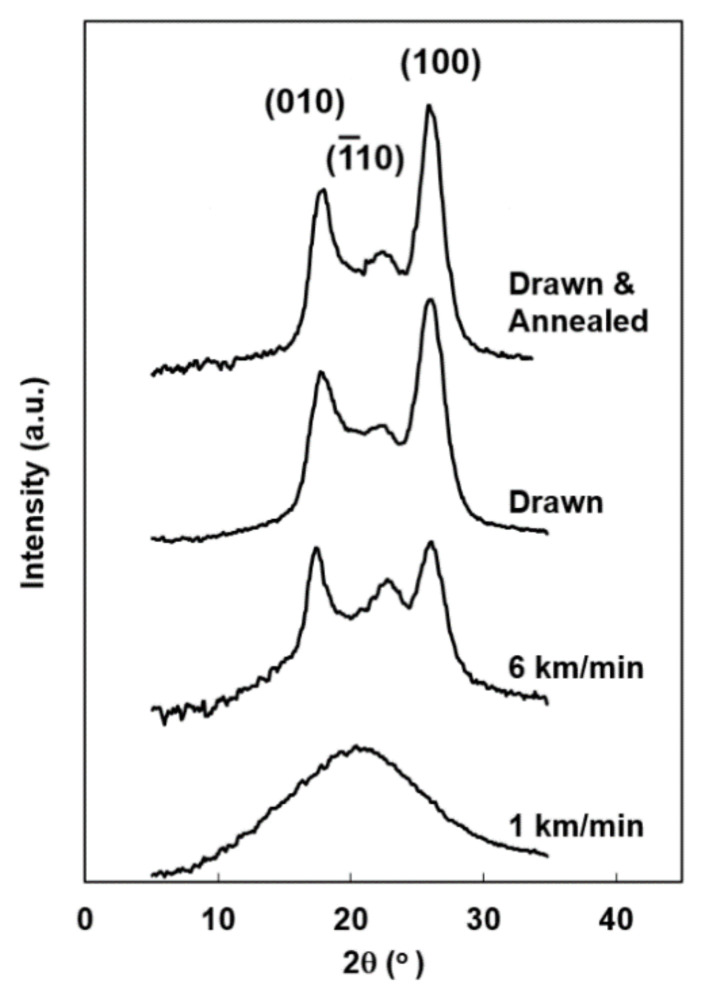
Equatorial WAXD intensity profiles for the high-speed spun H-PEF fibers obtained at take-up velocities of 1.0 and 6.0 km/min. Intensity profiles of drawn fiber and drawn and annealed fiber are also shown for comparison.

The equatorial intensity profiles of the H-PEF fiber prepared at 6.0 km/min, the drawn fiber, and the drawn and annealed fiber are compared in Figure 6, enabling the analysis of the crystalline structure of the PEF fibers in detail. The tree distinct crystalline peaks from low to high diffraction angles can be assigned to the (010), $\left(\bar{1}10\right)$, and (100) reflections, respectively. Crystalline reflection intensities were lower in with the intensity of amorphous halo for the high-speed spun fibers. The half-height widths of (010) and (100) for the high-speed spun fiber were 1.6 and 1.8 degrees, respectively, whereas those for the drawn fiber and the drawn and annealed fiber were 2.1 and 2.1 degrees and 1.8 and 2.0 degrees, respectively. The crystallite size increased with the application of annealing, whereas the high-speed spun fiber was consisting of larger crystallites even though its crystallinity was relatively low.

**Figure 7:**
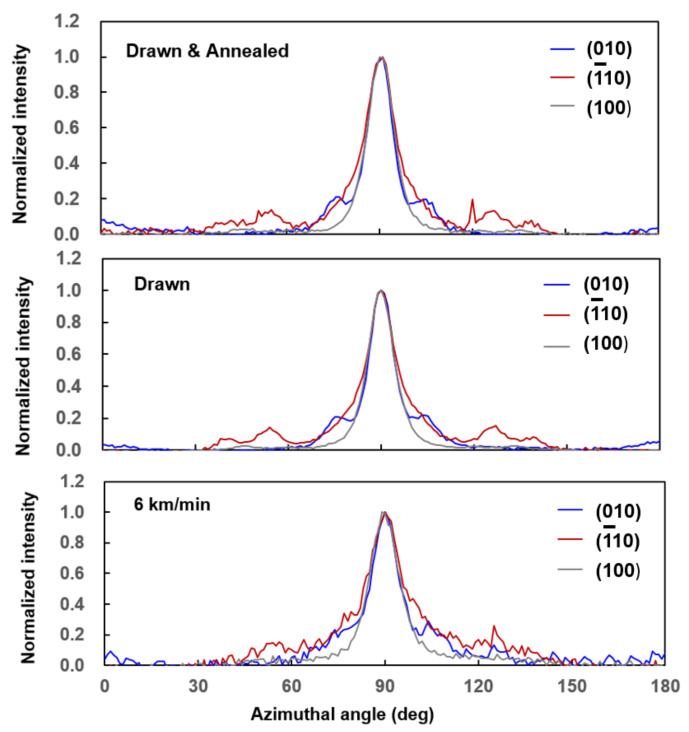
Azimuthal WAXD intensity distributions for (010), $\left(\bar{1}10\right)$, and (100) reflections of high-speed spun H-PEF fiber obtained at 6.0 km/min. Results for drawn fiber and drawn and annealed fiber are also shown for comparison.

A careful study on the relative peak intensity from each crystalline plane revealed that the intensity ratio of the three reflections varied depending on the processing conditions. Especially, the intensity for $\left(\bar{1}10\right)$ was high for the high-speed spun fiber. There can be two possibilities for the origin of such difference: one is the higher structural order in the direction normal to $\left(\bar{1}10\right)$ plane, and the other is the lower tilting angle of the *c*-axis with respect to the fiber axis for the high-speed spun fibers. It should be noted that the occurrence of the tilting of the *c*-axis along $\left(\bar{2}30\right)$ is well known for the crystalline structure of the PET fibers [30], where the high-speed spun fibers are reported to show a lower tilting angle than the conventional drawn and annealed fibers [31]. Azimuthal intensity profiles for the three distinct equatorial reflections, (010), $\left(\bar{1}10\right)$, and (100), are compared in Figure 7. The intensity profiles overlapped each other quite well for the three types of fibers. This means that there is no indication of tilting of the crystalline *c*-axis with respect to the fiber axis, and the *c*-axis orients parallel to the fiber axis in the PEF fibers. These results also suggested the higher ordering of crystalline structure in the direction perpendicular to the $\left(\bar{1}10\right)$ for the high-speed spun PEF fibers [32,33].

### 3.3. Mechanical Properties

**Figure 8:**
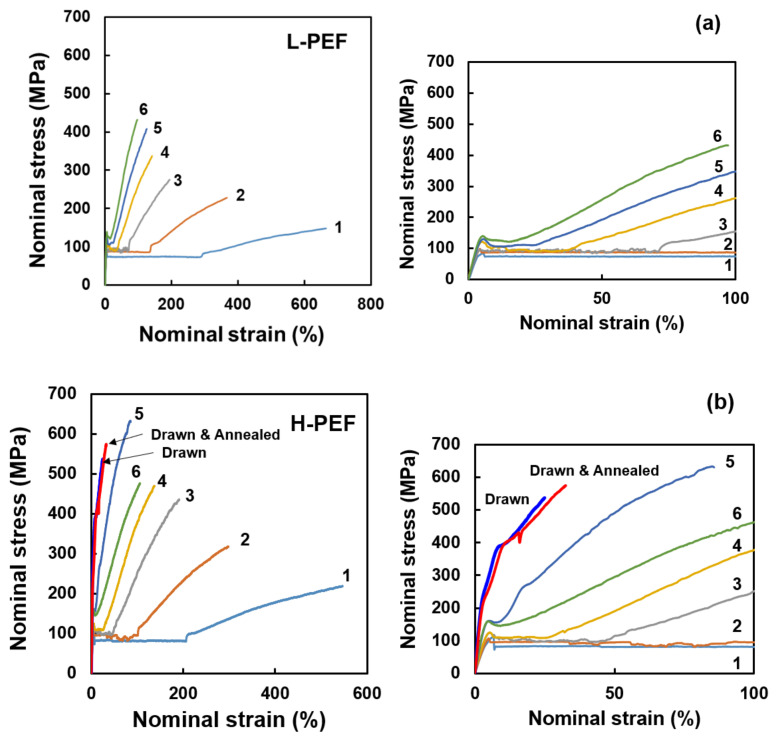
Stress–strain (s–s) curves of high-speed spun (**a**) L-PEF and (**b**) H-PEF fibers obtained at various take-up velocities. The results for the drawn and the drawn and annealed fibers are included for comparison. Enlarged s–s curves for the initial stage of stretching are also shown.

Typical stress–strain curves of the as-spun L-PEF and H-PEF fibers of various take-up velocities are shown in Figure 8a,b. Enlarged stress–strain curves for the initial stage of stretching are inserted in the figure. In general, tenacity increased and elongation at break decreased with the increase of take-up velocity. The H-PEF fibers showed higher tenacity and lower elongation than the L-PEF fibers as expected from the result of birefringence measurement shown in Figure 4a. At the initial stage of elongation, a region with constant stress was observed after the yielding. This behavior indicates the occurrence of necking deformation during the stretching. The draw ratio where the stress starts to increase again is called natural draw ratio. It is obvious that the natural draw ratio also decreased with the increase of take-up velocity, and the H-PEF fiber showed lower natural draw ratio than the L-PEF fiber at the same take-up velocity. For the L-PEF fibers, the occurrence of necking drawing was confirmed even for the fiber prepared at the take-up velocity of 6.0 km/min. For the H-PEF fiber, necking behavior was clearly observed up to 4.0 km/min. The fibers prepared at 5.0 and 6.0 km/min did not have the region of constant stress, yet still exhibited a distinct yielding behavior. These results suggested that the molecular orientation did not fully develop for the PEF fibers prepared even at high take-up velocities. The drawn fiber and the drawn and annealed fiber of H-PEF showed steeper increase of stress during stretching with a yielding at the stress of approximately 400 MPa, which resulted in the high strength and low elongation at break.

**Figure 9:**
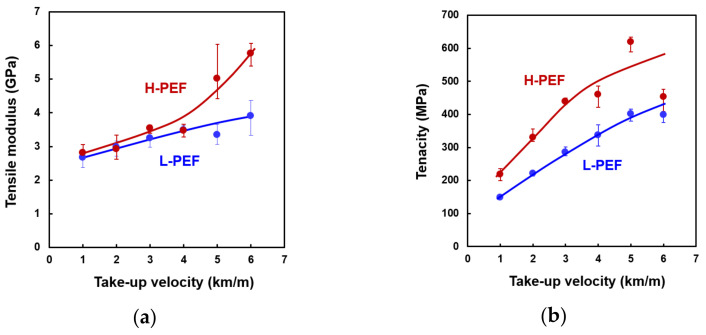

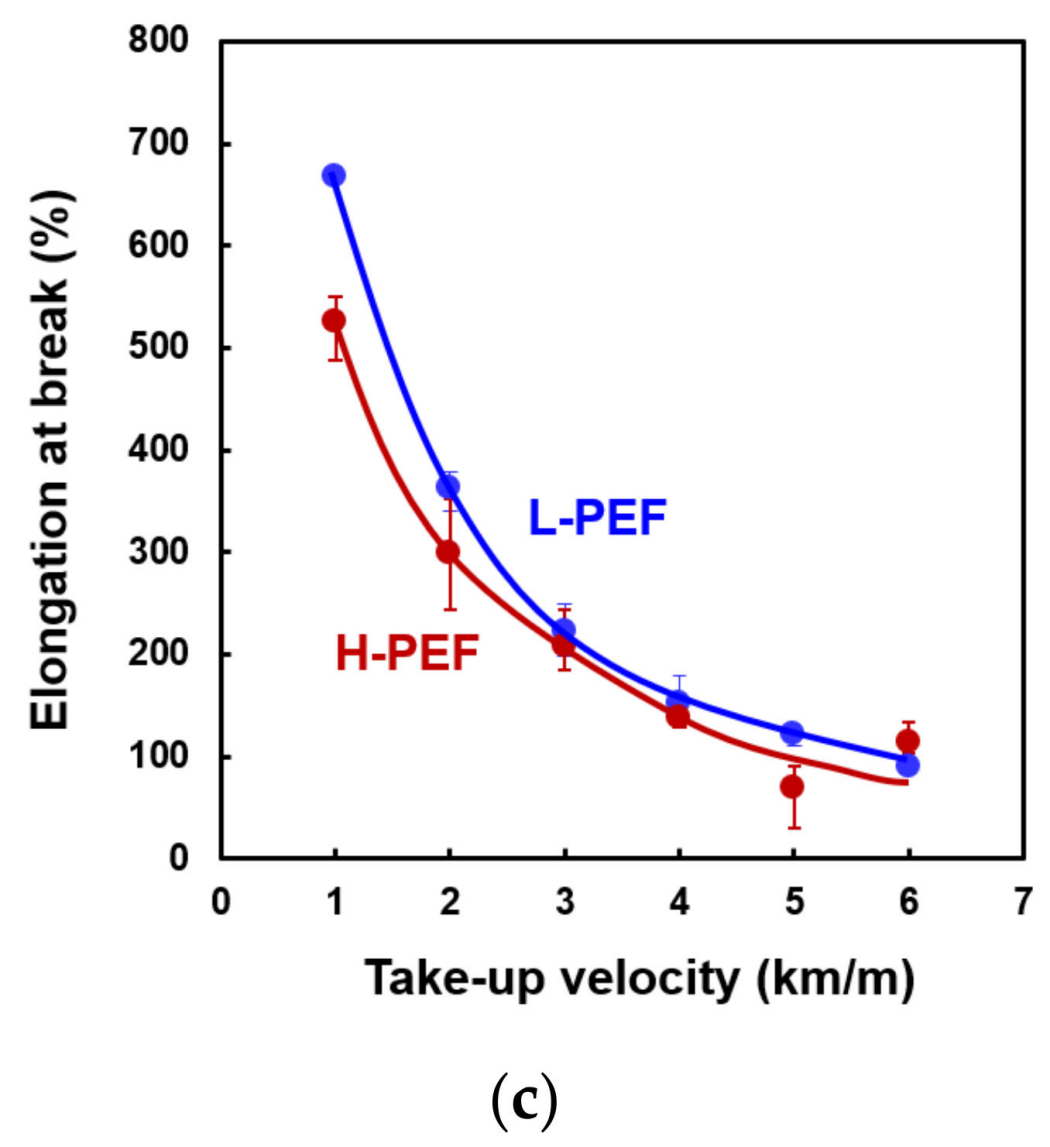
Variations of (**a**) tensile modulus, (**b**) tenacity, and (**c**) elongation at break with take-up velocity for high-speed spun L-PEF and H-PEF fibers.

The evolution in tensile modulus, tenacity, and elongation at break of the PEF fibers with the increase of take-up velocity are shown in Figure 9. In general, tensile modulus and tenacity increased whereas elongation at break decreased with the increase of take-up velocity. At the high-take-up velocities of 5.0 and 6.0 km/min, the tensile modulus and tenacity of the H-PEF fibers reached approximately 5 GPa and 500 MPa, respectively. The H-PEF fibers showed higher tensile modulus and tenacity, and lower elongation at break than the L-PEF fibers. These changes correspond to the above displayed status of the higher order structure of the high-speed spun PEF fibers.

Tensile modulus, tenacity, and elongation at break for the drawn fiber were 9.5 GPa, 533 MPa, and 29%, respectively, while those for the drawn and annealed fiber were 8.4 GPa, 550 MPa, and 31%, respectively. The high-speed spun PEF fibers exhibited significantly lower tensile modulus but almost comparable tenacity in comparison with the drawn fiber and the drawn and annealed fiber. It is well known that the low tensile modulus, which is caused by the low amorphous orientation, is a unique characteristic of high-speed spun PET fibers [27,29]. Tenacity of the high-speed spun PEF is equivalent or higher than that for high-speed spun PET fibers, however, tensile modulus was much lower even in comparison with that for the high-speed spun PET fibers.

**Figure 10:**
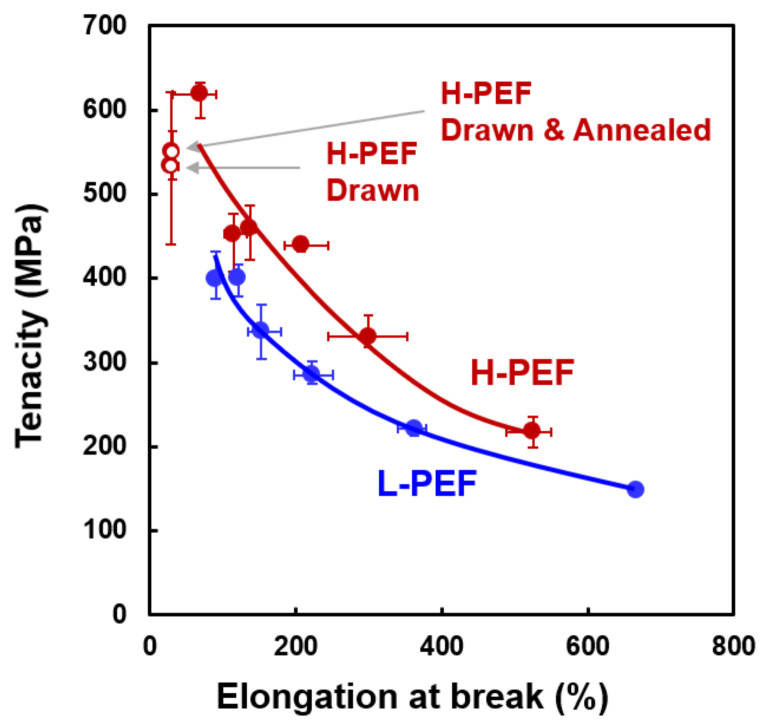
Relation between tenacity and elongation at break for high-speed spun L-PEF and H-PEF fibers. Data for drawn fiber and drawn and annealed fiber are also shown for comparison.

The relation between the tenacity and elongation at break for the H-PEF and L-PEF fibers are shown in Figure 10. Elongation at break decreased with the increase of tenacity, while the curve shifted to upper-right direction with the increase of molecular weight. This result generally indicates that the toughness of the high-speed spun fibers can be improved with an increase in the molecular weight.

## 4. Conclusions

High-speed spinning of high and low molecular weight PEF, (H-PEF and L-PEF) was performed. For both polymers, the take-up velocity of 6.0 km/min, the maximum velocity for producing PET fibers in the industry, could be attained. On-line measurement of the fiber diameter profile in the spin-line was performed, and the neck-like deformation was found to exist at high take-up velocities. The H-PEF fiber prepared at 6.0 km/min exhibited highly oriented crystalline reflections in the WAXD analysis, and the occurrence of the orientation-induced crystallization in the spin-line was confirmed. Birefringence and Lorentz-density of the as-spun fibers increased with the increase of take-up velocity, where H-PEF fibers showed higher molecular chain orientation and density than the L-PEF fibers. The mechanical properties of the resultant PEF continuous fibers were sufficiently high for a potential application in general semi-engineering field [34]. These results confirmed the applicability of PEF in fiber applications. Considering that PEF is an alternating bio-based polymer for PET, it also should be noted that similar fiber formation conditions for PET are applicable for producing of PEF fibers.

## Data Availability

Data sharing not applicable.
